# Deep Learning-Based Decision-Tree Classifier for COVID-19 Diagnosis From Chest X-ray Imaging

**DOI:** 10.3389/fmed.2020.00427

**Published:** 2020-07-14

**Authors:** Seung Hoon Yoo, Hui Geng, Tin Lok Chiu, Siu Ki Yu, Dae Chul Cho, Jin Heo, Min Sung Choi, Il Hyun Choi, Cong Cung Van, Nguen Viet Nhung, Byung Jun Min, Ho Lee

**Affiliations:** ^1^Medical Physics and Research Department, Hong Kong Sanatorium & Hospital, Happy Valley, Hong Kong; ^2^Artificial Intelligent Research Lab, Radisen, Seoul, South Korea; ^3^Vietnam National Lung Hospital, Hanoi, Vietnam; ^4^Department of Radiation Oncology, Chungbuk National University Hospital, Cheongju, South Korea; ^5^Department of Radiation Oncology, Gangnam Severance Hospital, Yonsei University College of Medicine, Seoul, South Korea

**Keywords:** chest X-ray radiography, COVID-19, deep learning, image classification, neural network, tuberculosis

## Abstract

The global pandemic of coronavirus disease 2019 (COVID-19) has resulted in an increased demand for testing, diagnosis, and treatment. Reverse transcription polymerase chain reaction (RT-PCR) is the definitive test for the diagnosis of COVID-19; however, chest X-ray radiography (CXR) is a fast, effective, and affordable test that identifies the possible COVID-19-related pneumonia. This study investigates the feasibility of using a deep learning-based decision-tree classifier for detecting COVID-19 from CXR images. The proposed classifier comprises three binary decision trees, each trained by a deep learning model with convolution neural network based on the PyTorch frame. The first decision tree classifies the CXR images as normal or abnormal. The second tree identifies the abnormal images that contain signs of tuberculosis, whereas the third does the same for COVID-19. The accuracies of the first and second decision trees are 98 and 80%, respectively, whereas the average accuracy of the third decision tree is 95%. The proposed deep learning-based decision-tree classifier may be used in pre-screening patients to conduct triage and fast-track decision making before RT-PCR results are available.

## Introduction

Coronavirus disease 2019 (COVID-19) caused by severe acute respiratory syndrome coronavirus 2 (SARS-CoV-2) has spread from Wuhan to the rest of China and to several other countries since December 2019. More than 2 million cases were confirmed by April 18, 2020. Worldwide, more than 150,000 deaths due to COVID-19 have been reported^1^.

COVID-19 is typically confirmed by reverse transcription polymerase chain reaction (RT-PCR). However, the sensitivity of RT-PCR may not be high enough for early detection, complicating the treatment of presumptive patients (1, 2).

Chest radiography imaging such as X-ray or computed tomography (CT), which is a routine technique for diagnosing pneumonia, can be easily performed, and it provides a quick, highly sensitive diagnosis of COVID-19 (1). Chest X-ray (CXR) images show visual indexes associated with COVID-19 (3), and several studies have shown the feasibility of radiography as a detection tool for COVID-19 (4–8).

To date, there have been no detailed studies on the potential of artificial intelligence (AI) to detect COVID-19 automatically from X-ray or chest CT images due to the lack of availability of public images from COVID-19 patients. Recently, some researchers have collected a small dataset of COVID-19 CXR images to train AI models for automatic COVID-19 diagnosis^2^ (9). These images were taken from academic publications reporting the results of COVID-19 X-ray and CT imaging. Minaee et al. published a study on COVID-19 prediction in CXR imaging using transfer learning (10). They compared predictions of four popular deep convolutional neural networks (CNN), which are ResNet18, ResNet50, SqueezeNet, and DenseNet-161. They trained the models using COVID-19 and non-COVID datasets, including 14 subclasses containing normal images from the ChexPert dataset (11). The models showed an average specificity rate of ~90% with a sensitivity range of 97.5%. This strongly encourages the hope that COVID-19 can be distinguished from other diseases and normal lung conditions by CXR imaging.

Tuberculosis (TB) is the fifth leading cause of death worldwide, with ~10 million new cases and 1.5 million deaths every year (12). Since TB caused by the bacteria that most often affect the lungs can be cured and prevented, the World Health Organization recommends systematic and broad screening to eliminate the disease. Despite its low specificity and interpretational difficulty, posteroanterior (PA) chest radiography is one of the preferred TB screening methods. TB is primarily a disease of poor countries; therefore, clinical officers trained to interpret these CXRs are often lacking (13, 14).

Several computer-aided diagnosis (CAD) researches dealing with CXR abnormalities do not focus on other specific diseases (non-TB). Most CAD systems specializing in TB detection have been reported (15–20).

TB and non-TB images are mixed in actual examination of lung disease. For diagnostic purposes, images should be classified as normal, TB, or non-TB. Several studies have been conducted on the automatic detection of various kinds of lung disease, which are not restricted to TB (21–25). In particular, the detection of anomalies, including non-TB ones, has been investigated with a multi-CNN model (26). Using an algorithm that only learns TB and normal data can result in lower accuracy when applied to examinations involving non-TB images.

In this study, we propose a deep learning-based decision-tree classifier comprising three levels. Each decision tree is trained by a deep learning model with a PyTorch frame-based convolution neural network. Using the proposed classifier, we investigate whether the detection of COVID-19 is feasible in CXR images. Furthermore, we quantify the accuracy of detection rates for normal, TB, COVID-19, and other non-TB diseases to see if the proposed classifier is a good candidate for clinical purposes.

## Method

### Workflow

**Figure 1:**
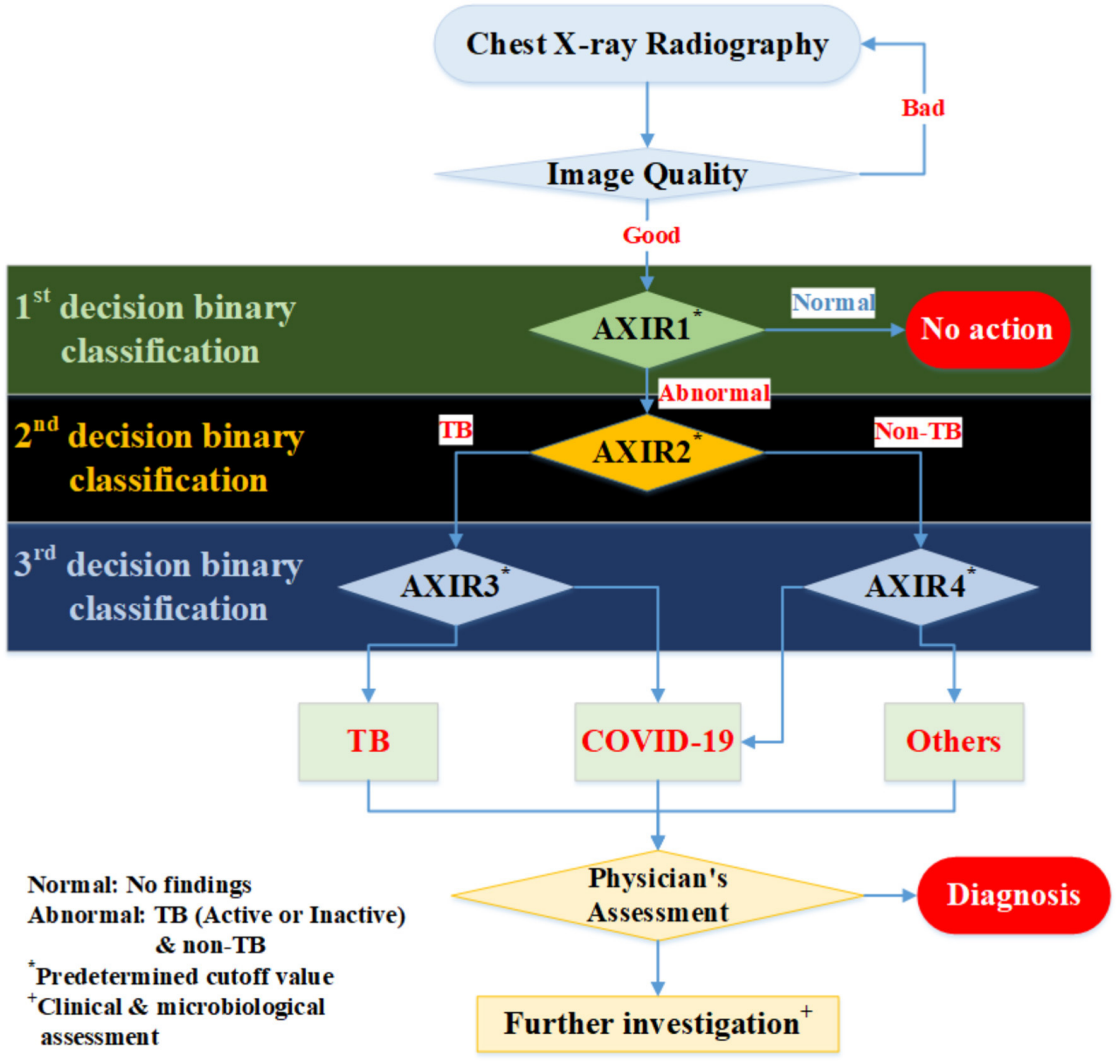
Workflow for determining whether chest X-ray image shows a normal, tubercular (TB), or COVID-19 infected lung. AXIR (Automated X-ray Imaging Radiography system).

In this study, we suggest a three-level decision-tree classifier for dividing CXR from the hospital or examination bus into four clinical states (normal, TB, non-TB, and COVID-19). Each step involves a binary classification, as shown in Figure 1. The image quality of each CXR is checked by the radiographer when it is taken, before the CAD model makes any predictions. In our suggested workflow, there are four automated X-ray imaging radiography systems (AXIRs). AXIR1 and AXIR2 are for the classification of images into abnormal/normal and TB/non-TB, respectively. If a good quality CXR is taken by AXIR1 and classified as an image of the normal state, no further action is taken. However, if the image is classified as abnormal, the next step (using AXIR2) is to determine whether it is TB or non-TB. AXIR3 and AXIR4 are used in the third step of the process, where the image is classified as COVID-19 or non-COVID-19, with or without TB. Finally, a medical doctor reviews the images and their classifications before making decisions about further investigation for clinical assessment.

In each step, the prediction model is trained with a data group optimized for its intended purpose. AXIR1 is trained with a combination of normal and abnormal data, the latter including both TB and non-TB data. The NIH ChestX-ray14 dataset was used in this study. AXIR2 is trained on TB and non-TB cases taken from the ChestX-ray14 dataset. In AXIR3, COVID-19 data, and non-COVID TB data are used for training, whereas in AXIR4, COVID-19, and non-COVID non-TB data are used. Each of the classifiers in the three-step system is designed for binary classification.

### Patient Data and Augmentation

In an effort to provide sufficient training data for the research community, allowing benchmark tests, the U.S. National Library of Medicine has made two datasets of posteroanterior (PA) chest radiographs available: the MC set and the Shenzhen set, Both datasets contain normal and abnormal chest X-rays with manifestations of TB and include associated radiologist readings. The categorization of TB is based on the final pathological diagnosis. The Shenzhen set was used in this study for TB dataset. The open-source Shenzen data comprise 326 TB and 226 normal cases (https://lhncbc.nlm.nih.gov/publication/pub9931). The average image size for Shenzen data is 3000 × 3000.

Also, the NIH (National Institutes of Health, US) Clinical Center recently released over 100,000 anonymized chest x-ray images and their corresponding data to the scientific community. The database from NIH is available online at https://nihcc.app.box.com/v/ChestXray-NIHCC/folder/36938765345. The size of each image is 1024 × 1024. This dataset is categorized into 14-lung disease sub-patterns which are based on pathological diagnosis. However, this dataset does not show TB or non-TB classification with pathological confirmation.

In addition to above open dataset, we used Eastern Asia Hospital dataset (which is based on cooperation with Radisen) for TB and non-TB disease. This dataset is based on pathological diagnosis. In the case of the non-TB data, NIH 14 based categorization was applied with pathological diagnosis. The images were taken by a Vicomed system and viewed with a Radisen Detector (17” × 17”). The average image size is 2484 × 3012. The image acquisition conditions were as follows: voltage = 105 kVp, current = 125 mA, charge = 10 mAs, time = 80 ms, source-to-image distance = 130–150 cm. For the correct collection of the data, two radiologists review dataset and only data which is agreed by two radiologists were used in this study.

Finally, we used the recently published COVID-Chest Xray-Dataset, which contains a set of images from publications on COVID-19 topics (7, 8). We used 162 images of COVID-19 infected lungs and transferred them all to image size 1,024 × 1,024 before training and testing. The original COVID-19 image sizes are various (not the same), because the images were taken from multiple institutions. Therefore, the different image sizes were normalized to 1,024 × 1,024, which is the smallest image size of the obtained images, to avoid image size effect on the performance. Moreover, COVID-19 dataset is based on pathological diagnosis (7–9)^2^.

In the case of the AXIR1, among the 1,170 patients' CXRs in the total dataset (Table 1), 85 normal and 85 abnormal CXR (total 170) scans were randomly selected from the data. The remaining 1,000 CXRs were split in a 50:50% ratio to make abnormal (500 patients) and normal (500 patients) cases for training. Among the 585 abnormal cases, 442 were from NIH data and 143 were from East Asian hospital data. The normal data images also came from the NIH and East Asian countries' data.

**Table 1:** Data structure for AXIR1.

**Normal/abnormal**	**Data group**	**Number of images**	**Sub total**
Abnormal	NIH data (TB + non-TB)^a^	442	585 (Train 500, Test 85) (Train with augmentation 1500)
	East Asian Hospital data (TB + non-TB)^b^	143	
Normal (NIH data + East Asian Hospital data)		585 (Train 500, Test 85) (Train with augmentation 1500)	

a *TB and non-TB are not classified with pathological diagnosis*.

b *TB and non-TB are classified with pathological diagnosis*.

In the case of the AXIR2, among the 984 patients in the total dataset (Table 2), 164 CXR scans (16.7%) were randomly selected for testing. Among these 164 CXR images, 82 were TB cases, and 82 were non-TB (other) cases. The remaining 820 images were split in a 50:50% ratio to make TB (410 patients) and non-TB cases (410 patients) for training. Among the 492 TB patients' images, 372 images were from East Asian data, and the remaining 120 TB cases were from Shenzhen data. All 492 non-TB lung disease images were taken from East Asian hospital data.

**Table 2:** Data structure for AXIR2.

**TB/non-TB**	**Data group**	**Number of images**	**Sub total**
TB	TB (East Asian Hospital data)	372	492 (Train 410, Test 82) (Train with augmentation 1500)
	TB (Sehnzen data)	120	
Non-TB (East Asian Hospital data)		492 (Train 410, Test 82) (Train with augmentation 1500)	

In the case of the AXIR3, of the 324 patients' CXRs in the total dataset (Table 3), 84 images (23.0%) were randomly selected for testing. Among these 84 images, 42 were COVID-19 cases and 42 were TB (non-COVID) cases. The remaining 240 scans were split in a 50:50% ratio to make COVID-19 (120 patients) and TB (non-COVID) cases (120 patients) for training.

**Table 3:** Data structure for AXIR3.

**Deep learning algorithm**	**COVID-19/TB**	**Training (Augmentation)**	**Test**
PyTorch, Resnet18 model, epoc = 50, 256*256 image size	COVID-19 (GitHub)	120 (608)	42
	TB (Shenzen data)	120 (608)	42

In the case of the AXIR4, among the 324 patients' CXRs in the total dataset (Table 4), 84 images (23.0%) were randomly selected for testing. Of these 84 images, 42 were COVID-19 cases and 42 were non-TB (non-COVID) cases. The remaining 240 scans were split in a 50:50% ratio to make COVID-19 (120 patients) and others (non-COVID) cases (120 patients) for training.

**Table 4:** Data structure for AXIR4.

**Deep learning algorithm**	**COVID-19/others**	**Training (Augmentation)**	**Test**
PyTorch, Resnet18 model, epoc = 50, 256*256 image size	COVID-19 (GitHub)	120 (608)	42
	Non-TB (Eastern Asian Hospital)	120 (608)	42

We applied several data augmentation algorithms to improve the training and classification accuracy of the CNN model and achieved remarkable validation accuracy. For AXIR1, 1,000 images were used for the augmentation of 3,000 images (3 times). For AXIR2, 820 images were used to augment 3,000 images (3.7 times). For AXIR3 and AXIR4, 240 images were used for the augmentation of 1,216 images (5 times). We applied the augmentation method as shown in Table 5. During training, the images were randomly rotated by 10°, translated, and horizontally flipped. In some cases, two methods (translation and rotation) were used at the same time.

**Table 5:** Settings for the image augmentation of training data.

**Method**	**Setting**
Rotation angle	10
Width shift	0.2
Height shift	0.2
Horizontal flip	True

### Annotation and Classification

We further categorized abnormal patterns of the NIH data as TB or non-TB. TB patterns were then subcategorized into seven active TB pattern classes (consolidation, cavitation, pleural effusion, miliary, interstitial, tree in bud, and lymphadenopathy) and an additional inactive TB pattern class. These TB pattern classes are based on those used by the Centers for Disease Control and Prevention (CDC) of the USA (26, 27). Also, we subcategorized the Shenzhen TB data into sub-classes [according to the ChestX-ray14 dataset (NIH data)], which are infiltration, consolidation, pleural effusion, pneumonia, fibrosis, atelectasis, nodule, pneumothorax, pleural thickening, mass, hernia, cardiomegaly, edema, and emphysema. These additional categorizations are based on the radiological readings for the purpose of the detailed analysis for sub-patterns of false negative (FN) and false positive (FP). The resulting annotated data were reviewed by two radiologists independently. In the case of the TB data, most data are related with infiltration, consolidation, plural effusion, fibrosis, and nodule. These patterns are strongly related with TB patterns.

We also used East Asia countries' data (TB and non-TB disease) from our cooperating hospitals to diversify the trained dataset. Non-TB disease dataset for Easter Asia Hospital is categorized based on NIH-14 disease pattern related with final confirmed diagnosis. In the case of the TB data (Eastern Asian Hospital), we annotated more detailed sub-patterns according to radiological sub-TB patterns. However, these sub-TB patterns are categorized with only radiological readings.

### Deep Learning Model and Training Technique

**Figure 2:**
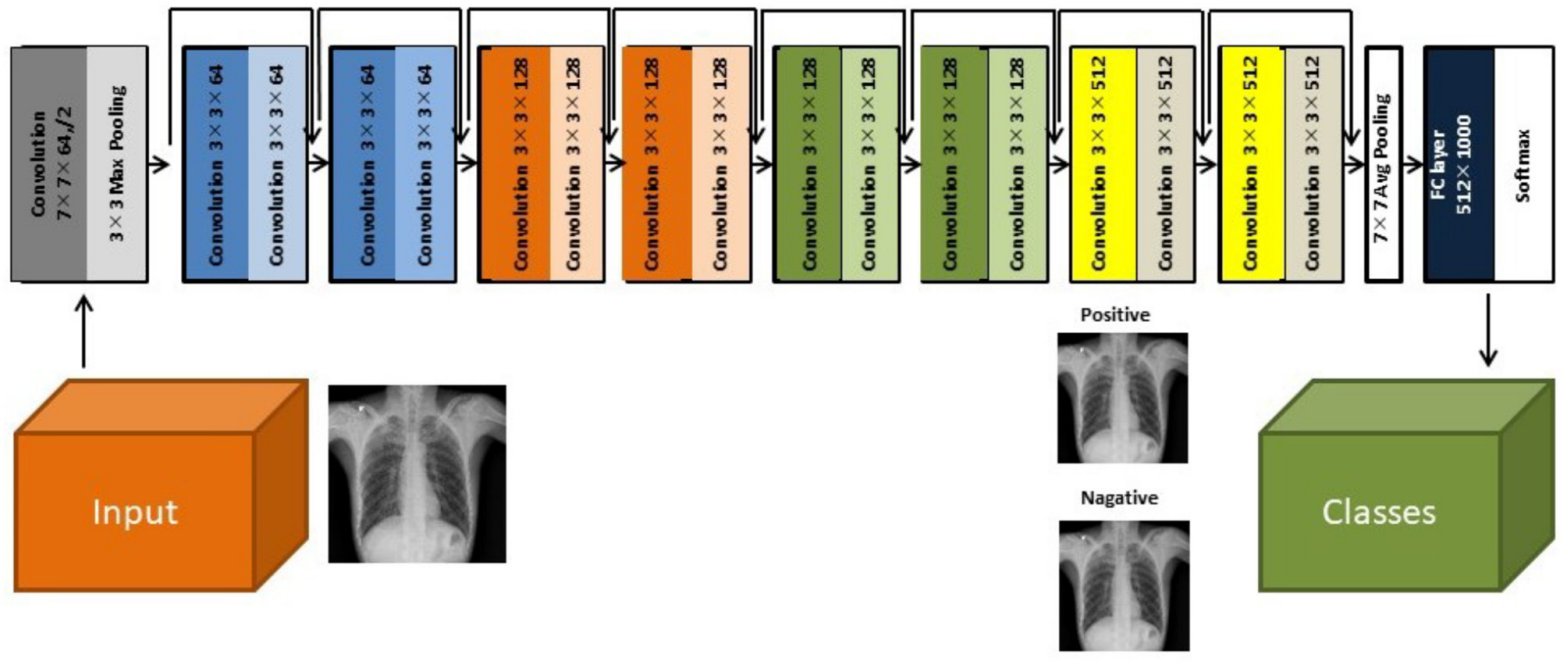
The architecture of the ResNet18 convolutional neural network model (28).

Herein, we used a two-dimensional CNN algorithm with a PyTorch frame for the training and testing for our two-step CAD process. Figure 2 shows the overall architecture of the proposed CNN model, which is based on the pre-trained ResNet18 (28, 29), using the ImageNet dataset. ResNet, the winner of the 2015 ImageNet competition, is one of the most popular CNN architectures, which provides easy gradient flow for more efficient training. The core idea of ResNet is the introduction of an “identity shortcut” connection, which skips one or more layers, thereby providing a direct path to the very early layers in the network, making the gradient updates for those layers much easier.

The verification of the model has been performed using an additional dataset. The performance of the model ResNet18 can be compared with previous work on neural nets and COVID-19 (9, 28) and verified as good matching with therein.

## Results

### Accuracy of AXIR1 and AXIR2 (Identification of Normal, TB, and Non-TB Patterns)

The performances attained by AXIR1 and AXIR2 are listed in Table 6. The accuracies of the two decision trees were 0.98 and 0.80, respectively.

**Table 6:** Classification performance of AXIR1 (Normal/Abnormal) and AXIR2 (TB/non-TB).

**AXIR**	**Accuracy**	**Sensitivity**	**Specificity**	**Precision**	**AUC**
AXIR1 (Step 1)	0.98	0.99	0.97	0.97	0.98
AXIR2 (Step 2)	0.80	0.72	0.89	0.87	0.80

*AUC, area under curve*.

Furthermore, we investigated the classification capability of AXIR2 for normality using 100 normal test data. The normal data can be classified into TB and others with the ratio of 1:3 by AXIR2.

In addition, we analyzed detailed distribution of false negative (FN) and false positive (FP) classification errors in AXIR2. The decision tree made 9 FP and 23 FN predictions, as shown in Table 7. Atelectasis and plural thickening (67% of all FPs) and infiltration, consolidation, and fibrosis (NIH classification) (83% of all FNs) are the categories most likely to be involved in such errors. These three sub-patterns can be categorized as consolidation sub-pattern in CDC sub-TB classification.

**Table 7:** Sub-patterns for false positive (FP) and false negative (FN) results in AXIR2.

**Prediction results**	**NIH classification^a^**	**Number of images**	**TB pattern in (30)^a^**	**Total number of images**
FP (from East Asian Hospital data)	Infiltration	2	Non-TB	9
	Consolidation	1		
	Atelectasis	3		
	Plural thickening	3		
FN (from Shenzhen data and East Asian Hospital data)	Infiltration	5	Consolidation	23
	Consolidation	8	Consolidation	
	Plural effusion	2	Plural effusion	
	Fibrosis	6	Consolidation	
	Nodule	2	Milliary	

a *Sub-patterns are classified with only radiological readings*.

### Accuracy of AXIR3 and AXIR4 (Identification of COVID-19 and Non-COVID Patterns)

Table 8 shows the classification performance of AXIR3 and AXIR4. In the case of the AXIR3 (COVID-19/TB classification), all test data are classified correctly, and the accuracy is 100%. In the case of the AXIR4 (COVID-19/non-TB classification), accuracy was only 0.89 due to low specificity. Therefore, the average value of accuracy over both trees was 0.95, and the average values of sensitivity and specificity were 0.97 and 0.93, respectively.

**Table 8:** Classification performance of AXIR3 (COVID-19/TB) and AXIR4 (COVID-19/non-TB).

**AXIR**	**Accuracy**	**Sensitivity**	**Specificity**	**Precision**	**AUC**
AXIR3	1.00	1.00	1.00	1.00	1.00
AXIR4	0.89	0.93	0.86	0.87	0.89
Average	0.95	0.97	0.93	0.94	0.95

*AUC, area under curve*.

We tested AXIR1 with 42 COVID-19 test data. Only one COVID-19 image was predicted as normal (FN) by AXIR1. Also, we investigated the classification results of 42 COVID-19 test data in AXIR2. COVID-19 data were classified by AXIR2 as TB and other in the ratio 4:1.

**Figure 3:**
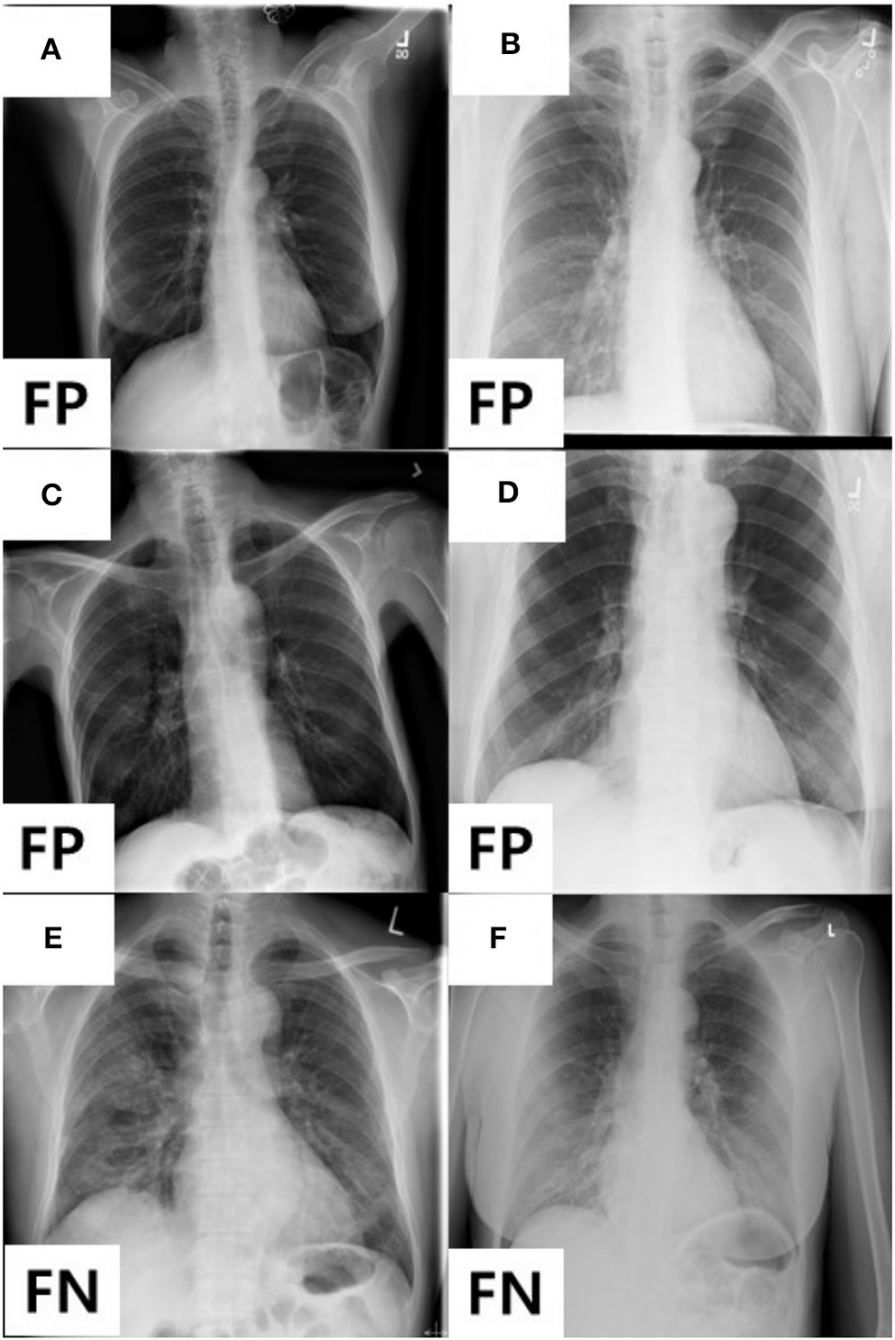
Images showing four FPs predicted as COVID-19 and two FNs predicted as non-TB lung disease in AXIR4 results: infiltration **(A–C)**, pleural thickening **(D)**, and COVID-19 **(E, F)** symptoms.

In addition, we analyzed the detailed distribution of FN and FP results in AXIR4. There are six FPs (annotated as non-TB (non-COVID) but predicted as COVID-19) and three FNs [annotated as COVID-19 but predicted as non-TB (non-COVID)]. Figure 3 shows the images that produced these results. Two of the three FNs were predicted as TB by AXIR2, whereas one image was predicted as non-TB.

## Discussion

The accuracy of AXIR1 (i.e., the binary decision-tree for classification of images as normal or abnormal) is 0.98. This prediction result shows that the ResNet18 model can be used clinically for the screening of abnormality. However, the accuracy of AXIR2 (i.e., the binary decision-tree for classification of images as showing TB or a different disease) is much lower (0.80), as shown in Table 6. The sensitivity and specificity for AXIR2 are 0.72 and 0.89 respectively. Table 7 shows the detailed distribution among these classes for 9 FP and 23 FN predictions of AXIR2. Atelectasis and plural thickening show a higher portion of FP (67%) and infiltration, consolidation and fibrosis are higher potion for FN (83%). In atelectasis and plural thickening, the region can be similar to plural effusion in lower lobe in both side of lung. Thus, the algorithm's prediction results can show larger portion of FP due to atelectasis and plural thickening specifically. Clinically, plural effusion is strongly associated with TB, but atelectasis or plural thickening is not. Moreover, infiltration, consolidation and fibrosis, as defined by the NIH, are strongly associated with TB; however, some of these radiological sub-patterns can be predicted as non-TB. When these sub-patterns are annotated as TB, the position of occurrence was frequently in the upper apex region. The most of cases of FP show that the locations of occurrence are middle or lower lobe area. TB screening is not only limited to the normal/abnormal classification, but can also locate its location in CXR with abnormalities (30, 31). The clavicle region is known to be a difficult area to detect TB because it can obscure the manifestations of TB at the apex of the lung. The automatic suppression of the ribs and clavicles in the CXR could significantly improve the decision of radiologists in the detection of nodule (32, 33). Therefore, applying position-based feature filtering in the algorithm might improve the accuracy. For the analysis of sub-pattern, the data were categorized with only radiological readings. However, all training data structure for the prediction results of TB and non-TB is based on pathological diagnosis.

We analyzed the performance of the classification into COVID-19 and non-COVID through the binary decision trees AXIR3 and AXIR4. We tested AXIR1 and AXIR2 with COVID-19 images. Among the COVID-19 test images, 98% (41 images of 42) were predicted as abnormal in AXIR1. Also, 80% of the COVID-19 images were predicted as TB in AXIR2. However, we tested the AXIR3 and AXIR4 step using all the 42 COVID-19 images for each AXIR. Table 8 shows that the accuracy of AXIR3 is 100%, although the accuracy of AXIR4 is only 89%. The average sensitivity (97%) is almost same as the 98% reported in (9), whereas the average specificity (93%) is slightly higher than 89%. We think that this is because normal images were excluded already by AXIR1. Also, if we recall that some FN images in AXIR4 (annotated as COVID-19) were categorized as TB in AXIR2, then those images are predicted as COVID-19 in AXIR3, and the accuracy could be improved in the real process.

Both COVID-19 and TB cause respiratory symptoms (cough and shortness of breath). One of the biggest differences is the speed of onset. TB symptoms do not tend to occur immediately after infection, but gradually appear, unlike COVID-19, which can occur within a few days. In this regard, we have combined the detection of TB or COVID-19.

There are practical considerations that require further investigation. First, this study used a suitable data group as training data for each step, but training data should be confirmed with pathological data. Without the use of pathologically confirmed data, the results of the model are unreliable. Therefore, prediction of new cases requires new pathological data. Second, the proposed three-level decision tree classifier takes three times longer than a one-step process using multiple classifiers. Third, there are many data augmentation strategies for image data. We added more training data to our deep learning model using easy-to-implement methods such as horizontal flip, rotations, and shifts. Image processing techniques using stochastic region sharpening, elastic transforms, randomly erasing patches, and many more to augment data can be considered to improve the performance of the resulting model. Further studies are thus needed on advanced augmentation techniques for building better models and creating a system that does not require gathering a lot of training data to get a reliable statistical model.

## Conclusions

Herein, a deep learning-based three-level decision-tree classifier for detecting TB and non-TB lung diseases, including COVID-19, has been presented and validated using patient data. For each level, a two-dimensional CNN algorithm (ResNet18 model) with PyTorch frame was used with optimized trained data. Accuracies of 98 and 80%, respectively, were achieved for AXIR1 (abnormal vs. normal data) and AXIR2 (TB vs. non-TB data). The lower accuracy of AXIR2 is due to FP atelectasis and plural thickening predictions and FN infiltration, consolidation, and infiltration. An average accuracy of 95% was achieved with AXIR3 (COVID-19 vs. TB) and AXIR4 (COVID-19 vs. other non-TB). We believe that this study will have significant clinical applications, allowing fast follow-up decision making and pre-screening in suspected COVID-19 cases prior to the availability of RT-PCR results.

## Data Availability Statement

The data analyzed in this study is subject to the following licenses/restrictions: data sets cannot be made available to the public. Requests to access these datasets should be directed to SYo, yoo7311@gmail.com.

## Author Contributions

Conception, design, and drafting the manuscript were performed by SYo, BM, and HL. Data collection was performed by DC, JH, MC, and IC. The statistical analysis was performed by HG, TC, and SYu. CC and NN interpreted the results. All authors read and approved the final manuscript.

## Conflict of Interest

The authors declare that the research was conducted in the absence of any commercial or financial relationships that could be construed as a potential conflict of interest.

## Footnotes

^1^https://www.worldometers.info/coronavirus/

^2^https://github.com/ieee8023/covid-chestxray-dataset
